# Effects of bradykinin preconditioning in an experimental intestinal ischemia reperfusion model on rats ^1^

**DOI:** 10.1590/s0102-865020200040000002

**Published:** 2020-06-19

**Authors:** Fatih Dal, Can Küçük, Tutkun Talih, Erdoğan Sözüer, Uğur Topal, Kemal Deniz, Hızır Akyıldız

**Affiliations:** I MD, Department of General Surgery, Erciyes University Medical Faculty, Melikgazi, Kayseri, Turkey. Conceptıon and design of the study; acquisition, analysis and interpretation of data; technical procedures; statistics analysis; manuscript preparation and writing; final approval.; II MD, Department of General Surgery, Erciyes University Medical Faculty, Melikgazi, Kayseri, Turkey. Conceptıon and design of the study, technical procedures, statistics analysis, manuscript preparation, critical revision.; III MD, Department of General Surgery, Erciyes University Medical Faculty, Melikgazi, Kayseri, Turkey. Analysis and interpretation of data, statistics analysis.; IV MD, Department of General Surgery, Erciyes University Medical Faculty, Melikgazi, Kayseri, Turkey. Conceptıon and design of the study, technical procedures, manuscrıpt wrıtıng, critical revision.; V MD, Department of General Surgery, Erciyes University Medical Faculty, Melikgazi, Kayseri, Turkey. Manuscript preparation and writing, final approval.; VI MD, Department of Surgical Pathology, Erciyes University Medical Faculty, Melikgazi, Kayseri, Turkey. Acquisition, analysis and interpretation of data; histopathological examinations.; VII MD, Department of General Surgery, Erciyes University Medical Faculty, Melikgazi, Kayseri, Turkey. Conceptıon and design of the study, technical procedures, manuscript writing, critical revision.

**Keywords:** Reperfusion Injury, Bradykinin, Ischemic Preconditioning, Intestine, Small, Rats

## Abstract

**Purpose:**

To investigate the effects of bradykinin on reperfusion injury in an experimental intestinal ischemia reperfusion model.

**Methods:**

We used 32 Wistar-Albino rats. We composed 4 groups each containing 8 rats. Rats in sham group were sacrified at 100 minutes observation after laparotomy. Thirty minutes reperfusion was performed following 50 minutes ischaemia in control group after observing 20 minutes. Ischaemic preconditioning was performed in one group of the study. We performed the other study group pharmacologic preconditioning by infusional administration of 10 μg/kg/minute bradykinin intravenously. We sacrified all of the rats by taking blood samples to evaluate the lactate and lactate dehydrogenase (LDH) after resection of jejunum for detecting tissue myeloperoxidase (MPO) activity.

**Results:**

Lactate and LDH levels were significantly higher in control and study groups than the sham group (P<0.001). There is no difference between the study groups statistically. (P>0.05). The results were the same for MPO levels. Although definitive cell damage was determinated in the control group by hystopatological evaluation, the damage in the study groups observed was lower in different levels. However, there was no significant difference between the study groups statistically (P>0.05).

**Conclusion:**

Either ischeamic preconditioning or pharmacologic preconditioning made by bradykinin reduced the ischemia reperfusion injury at jejunum.

## Introduction

Restoring blood flow to ischemic tissue is essential for energy supply and cell survival. The most important condition to reduce the progression of ischemic damage is to minimize its duration, but through the reperfusion of ischemic tissues, a series of complex reactions that initiate tissue damage have already begun^1^ . Parks and Granger showed for the first time in 1983 that reperfusion damage was more severe than ischemic damage^2^ .

The pathogenesis of ischemia reperfusion (IR) is due to both the depletion of cellular energy sources due to tissue hypoxia during ischemia and, more importantly, to the formation of O2-derived free radicals during reperfusion. The IR process can cause dysfunction and damage not only to the organ in which it is involved but also to distant organs through a range of mediators^3^ .

Recently, a surgical method known as ischemic preconditioning (IPC) has been developed to prevent reperfusion injury. The aim of this method is to increase resistance to IR damage in the organ with short ischemia and reperfusion episodes applied before long ischemia. The researchers aimed to establish a larger infarct area in the canine heart muscle, but observed that several short ischemia and reperfusion episodes that they added before long ischemia did not expand the infarction, but instead regressed it by 75%^4 - 6^ .

Pharmacological preconditioning (PPC), known to have protective effects against IR damage, similar to IPC, has been studied with many agents thought to be effective in the intestinal tract by different mechanisms^7 - 9^ .

Kallikrein Kinin System (KKS) is a system of kallikreins, kininogens, kinins, kinin degrading enzymes and kinin receptors^10^ . The kinin family consists of bradykinin (BK), kallidine, methionyllysyl-BK^11^ . Kinins, which have a broad spectrum of activity, are potent vasodilators. They play a protective role in IR injury by reducing post-ischemic leukocyte adherence to the endothelium, disruption of microvascular barriers and tissue damage^12^ .

In our study, we aimed to use BK, an endogenous vasodilator peptide as a pharmacological preconditioning agent in the intestinal ischemia reperfusion model.

## Methods

### Animals and preoperative preparation

This experimental study was conducted at the Hakan Çetinsaya Experimental and Clinical Research Center (DEKAM) of Erciyes University Faculty of Medicine between November and December 2007 with the approval of the Ethics Committee. (Ethics Committee Decision Date: 07.11.2006, Decision No: 399).

A total of 32 female Wistar albino rats, weighing between 250 and 300 g, were used for this study. The rats, which were kept in rooms without windows, were housed and observed in constant-temperature conditions and were fed with standard rat chow until the experiment day. The animals were randomized into four groups:

**Group I. Sham Group (n=8):** In this group, only laparotomy was performed on the subjects and they were observed for 100 minutes.

**Group II. Control Group (n=8):** In this group, ischemia reperfusion injury was created by 20 min of observation after laparotomy and 50 min ischemia by clamping of the **superior mesenteric artery** (SMA), followed by 30 min of reperfusion by opening the clamp

**Group III. IPC (Ischemic Preconditioning) Group (n=8):** Subjects in the IPC group received 10 min of ischemia, followed by 10 min of reperfusion, and then 50 min of ischemia and 30 min of reperfusion.

**Figure 1:**
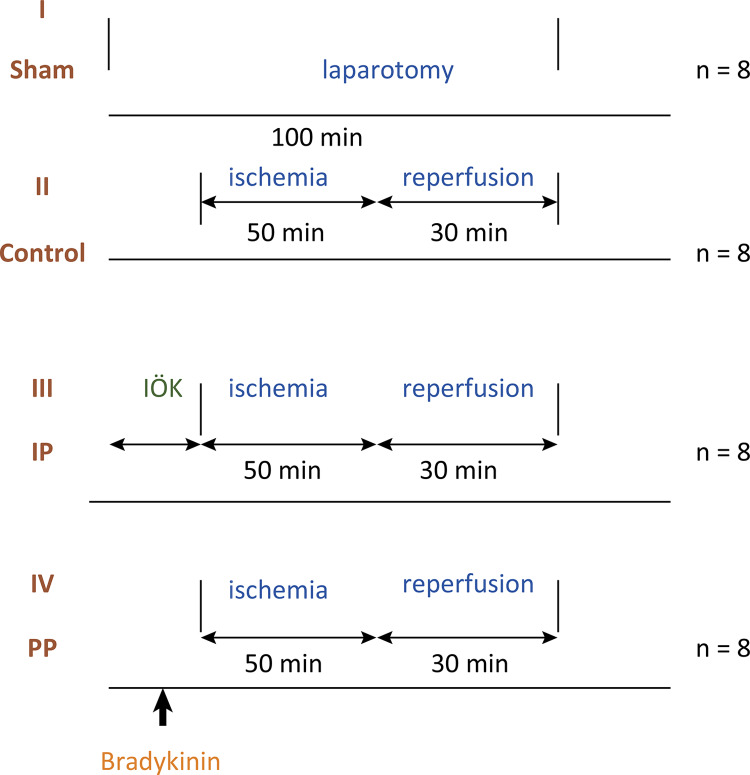
- Study setup.

**Group IV. PPC (Pharmacological Preconditioning) Group (n=8):** The administration of bradykinin (Sigma B3259, Steinheim, Germany) at a dose of 10 μg/kg/min for 15 min via a jugular venous catheter to the PPC group was followed by 50 min of ischemia and 30 min of reperfusion. Study setup has shown Figure 1 .

### Operative technique

Surgical procedures, injections, and blood samples were taken in all rats under intraperitoneal anesthesia with 50mg/kg ketamine (Ketalar^®^, Pfizer-Istanbul) and 10mg/kg xylazine (Rompun®, Bayer-Istanbul). Right jugular vein catheterization was performed one hour before the operation in all groups except the sham group and fluid replacement (0.9% NaCl 10ml/kg/h) was performed before major surgical intervention.

Laparotomy was performed with a longitudinal incision of approximately 6 cm from the xiphoid. After the intestinal structures were deviated to the left, the SMA was clamped with a bulldog clamp from the exit point of the aorta. Previously, planned ischemia and reperfusion timing was performed for each group. Fluid replacement (0.9% NaCl 10 mg/kg/h) was performed in all rats during the operation.

Five ml of blood was collected from the vena cava inferior (VCI) after 100 minutes of observation in the sham group, and after reperfusion in the other groups.

Approximately 3 cm of jejunal segment 10 cm distal from the treitz ligament was resected for tissue sampling. 2 cm of the specimen was stored in 10% formaldehyde for histopathological evaluation. Remaining 1 cm was stored at -80°C in aluminum foil for the determination of tissue myeloperoxidase (MPO) activity.

There was no mortality during the study. Sacrifice was made by hemorrhage created through the transverse incision of abdominal aorta and vena cava after tissue and plasma sampling.

### Measurements

Blood samples taken to heparinized syringes on the day of the experiment for lactate levels were studied simultaneously (Rapidlab865, Bayer, Germany) on the blood gas measurement device. The samples were transported to the laboratory at + 4°C. The results were united in mmol /L.

One ml of 5 ml blood was taken into heparinized syringes and simultaneous lactate study was performed. The remaining blood sample was centrifuged for 5 minutes at 3000 rpm for biochemical evaluation and plasma was separated and stored at -80°C until working day.

Lactate Dehydrogenase (LDH) levels were measured in serum dissolved at room temperature on working day from blood sample stored at -80°C. LDH levels were measured on a Beckman Coulter LX20 (USA) auto-analyzer and were united as U / L.

The method defined by Grisham was used by enzyme determination^13^ . Accordingly, 300 mg of intestinal mucosa was homogenized with 5 ml of cold 0.02M EDTA. The homogenate was centrifuged at 20000 rpm for 15 min at +4°C to discard the supernatant. The pellet was dissolved and re-centrifuged in 0.05M potassium phosphate buffer containing 0.5% hexadecyl trimethyl ammonium bromide (HTAB) with an equal volume and a pH of 5.4. MPO was determined spectrophotometrically at 410nm wavelength in the supernatant. The results were evaluated as units per gram tissue.

The jejunum samples stored in 10% formalin were followed up histopathologically. 5-micron thick sections were taken from the specimens embedded in paraffin blocks and stained with hematoxylin and eosin stain. The stained preparations were evaluated under light microscope. In all histopathological evaluations, the pathologist did not know which group the preparation they evaluated belonged to.

Histopathological changes in the jejunum due to IR injury were evaluated according to the grading of Chiu *et al* .^3^ ( Chart 1 ).

**Chart 1:** Chiu histopathological damage scoring.

**Stage 0**	Normal mucosa
**Stage 1**	Development of subepithelial space, usually at the tip of the villus, with capillary congestion
**Stage 2**	Extension of the subepithelial space with moderate lifting of the epithelial layer from the lamina propria
**Stage 3**	Massive epithelial lifting at the top of the villi
**Stage 4**	Denuded villi with lamina propria, dilated capillaries exposed, increased cellularity of the lamina propria
**Stage 5**	Digestion and disintegration of the lamina propria, hemorrhage, and ulceration

In this study, we used serum lactate and LDH values as biochemical parameters. MPO activity was also studied at tissue level, which has been shown to be one of the markers of IR injury and originates from inflammatory leukocytes. Finally, experimental IR injury and the effects of ischemic and pharmacological preconditioning, which are suggested as protective mechanisms against it, were examined histopathologically at the intestinal tissue level.

### Statistical analysis

Data were shown as mean ± standard deviation (X ± SD) and/or median (min-max). Data were analyzed by SPSS 15.0 for Windows (Statistical Package for Social Sciences) package program. Shapiro-Wilk normality test was used to determine whether the data showed normal distribution. One-way analysis of variance was used to determine whether there was a difference between the groups for normal distribution variables. Multiple comparisons were made with Tukey test for homogeneous variance groups and Tamhane test for non-homogeneous variance groups. The Kruskal-Wallis test was used to determine whether there was a difference between the groups for the variable that did not show normal distribution. Multiple comparisons were made with Student-Newman-Keuls Test. P <0.05 was considered statistically significant.

## Results

When LDH, Lactate and MPO values were compared, significantly lower levels were found between the sham group and the three other groups (p<0.001). The LDH, lactate and MPO levels were significantly lower in the IPC and PPC groups compared to the control group (p<0.001), and there was no significant difference between the PPC and IPC groups (p>0.05) ( Table 1 ).

**Table 1:** - Comparison of Serum LDH, Lactate, MPO values and Chiu score.

	Sham(n=8)	Control(n=8)	IPC(n=8)	PP(n=8)	p
Lactate(mmol/L)	1.29±0.12^a^	2.21±0.28	1.55±0.15^b^	1.71±0.19^b^	<0.001
LDH(U/L)	412.4±178.1^a^	2107.8±386.2	904.6±221.1^b^	1161.5±230.7^b^	<0.001
MPO (U/gr tissue)	0.60 ± 0.12^a^	1.62 ± 0.13	0.93 ± 0.12^b^	1.07 ± 0.10^b^	<0.001
Chiu score	0.00 (0.00-0.50)^a^	4.50 (4.00-5.00)	3.00 (2.50-3.00)^b^	3.00 (3.00-4.00)^b^	<0.001

^a^ significantly lower than the other 3 groups. ^b^ significantly lower than the control group. Values are given as mean ± standard deviation (X ± SD) and median (minimum-maximum). LDH: Lactate dehydrogenase. MPO: Myeloperoxidase.

In the control group, lamina propria and villi lifting, capillary dilatation, increased cellularity in lamina propria, and occasional areas of digestion and loss of integrity, bleeding and ulceration in the lamina propria were observed. There was a significantly higher level between the control group and all other groups (p<0.001). However, there was no statistically significant difference between IPC and PPC groups (p>0.05) ( Table 1 ).

## Discussion

IR damage is a complex process involving O2-derived free radicals, cytokines, NO and PMNLs^7^ . IR damage may occur in the intestinal system in cases of necrotizing enterocolitis, inflammatory bowel diseases, free pedicled bowel flap, cardiopulmonary bypass, strangulated hernia and bowel transplantation^14^ .

The oxygen-free stay of the tissue during ischemia disrupts mitochondrial functions and causes the energy source, ATP, to be consumed. Lack of energy disrupts the intracellular and extracellular balances of ions such as H +, Na + and Ca ++. Maintaining cell volume and integrity becomes increasingly difficult. Reactive oxygen radicals, which increase rapidly as the environment oxygenates with reperfusion, increase the oxidative stress in the cell. In addition to reactive oxygen radicals from other inflammatory cells in the circulation and parenchyma, especially macrophages, proinflammatory cytokines such as TNF – a, IL – 6, IL – 1b and IL – 12 are released into the environment. Reperfusion injury can induce organ failure by inducing the inflammatory response in distant organs through cytokines as well as locally^15^ .

For the past 15 years, efforts to prevent IR damage in the small intestine and to form new strategies have been intense. Various therapeutic modalities have been successfully used to protect tissue from IR damage in different animal models. These can be divided into: (a) Treatment with antioxidant agents, (b) NO applications, (c) Anticomplement therapy, (d) Treatment with perfluorocarbons, (e) Enteral nutrition, (f) Glycine and Glutamine application^7^ .

In the experimental intestinal IR model in rats, the duration of ischemia by obliteration of SMA and of reperfusion after obliteration is removed, is controversial. Mallick *et al* .^16^ used 30 min for ischemia and 120 min for reperfusion in their experimental model. In a study investigating the role of NO in IPC, Vlasov *et al* .^17^ established the damage model on 90 min ischemia and 30 min reperfusion. In the intestinal IR model of our study, we used the 50 min ischemia and 30 min reperfusion protocol previously used by Abrahao *et al* .^18^ , where all stages of reperfusion injury can be demonstrated histopathologically.

To date, many experimental studies have been planned to protect organs against IR damage using different surgical, pharmacological and genetic methods. Few studies that seemed to be successful in the experimental setting have had the chance to be practiced in the clinic. The role of IR damage being too complex to be prevented by blocking a single step or mediator is quite significant in this issue.

The protective effects of IPC were first described in the small intestine by Hotter in 1996, and since then, studies have gained intensity^19^ . According to this procedure, after an ischemic interval of 5-20 minutes by clamping the SMA with an occlusive vascular clamp, the clamp is removed, and reperfusion is achieved for a period of 5-15 minutes. The protective role of IPC against reperfusion damage in rat’s small bowel allografts results from its anti-inflammatory effect^20^ .

Another strategy to protect the small intestine from IR injury is preconditioning with pharmacological agents. In recent years, dozens of agents have been used in rat models, which are effective through different mechanisms. Antioxidants come into prominence in this context because the effect of free radicals is one of the major causes of reperfusion injury. Some of these are; allopurinol, SOD, deferoxamine, N-acetyl cysteine, ethanol, ascorbic acid, tocopherol, pentoxifylline, captopril and verapamil^7 , 21^ .

We used BK as a pharmacological preconditioning agent in this experimental study. The main protective effect of BK against intestinal IR is through inflammatory leucocytes. It prevents the adhesion of activated neutrophils to the endothelium and plays a protective role against neutrophil mediated reperfusion damage. Additionally, it inhibits the increase of capillary permeability by decreasing microvascular barrier deterioration^12^ .

Nilsson *et al* .^22^ in their ischemia reperfusion studies related to adhesion molecules such as CD11 / CD8, which provide neutrophil adhesion, obtained positive biochemical and morphological parameters on the damage caused by preventing the formation of monoclonal antibodies such as IB4, hypochloric acid caused by neutrophils and N-chloramines that are more toxic.

Ariceta *et al* .^23^ used somatostatin as a cytoprotective agent in the rats in which they performed IR and stated that the drug reduced leukocyte infiltration and decreased the level of tissue myeloperoxidase in intestinal IR damage in rats given the drug.

The protective effect of BK against reperfusion damage in various organs has been shown in experimental studies. Yan-Feng *et al* .^24^ used BK in the spinal cord ischemic damage model they created in rats, and obtained positive histopathological and biochemical parameters. Similarly, Ping *et al* .^25^ investigated the preconditioning effect in rats with focal cerebral ischemia and showed that the preconditioning effect of BK had positive results on the damage. Liuba *et al* .^26^ also showed that the damage in post-ischemic arterial endothelial cells regressed with the use of BK using morphological parameters.

In this experimental study, we reviewed the various publications and selected serum lactate and LDH values as biochemical parameters in demonstrating the possible protective effects of both reperfusion injury and ischemic and pharmacological preconditioning. In addition, MPO activity caused by inflammatory leukocytes, which has been previously shown to be one of the indicators of IR damage and plays an important role in the pathogenesis of damage, was also studied at the tissue level.

Vejchapipat *et al* .^27^ observed that lactate is a reliable parameter in grading the damage caused by reperfusion following IPC regulation and ischemia. Accordingly, plasma lactate level also reflects intestinal damage and protection from IP and damage. These authors argued that NO is the underlying protective mechanism in IPC^27^ . Abrahao *et al* .^18^ reported a statistically significant decrease in plasma lactate levels compared to the IR group in the experimental rat models in which they caused IR damage in the small intestine.

We also obtained similar results in our study. There was a statistically significant difference between the sham group and our control and study groups in terms of plasma lactate levels (p <0.001).

Since LDH activity is found in the stoplasms of various cell types, it has been used as a sensitive nonspecific tissue damage indicator. Intestinal isoform of LDH catalyzes a reversible reaction that produces lactate from purulent material. When more than one type of tissue is damaged in one area of the organism, the dehydrogenase activity in the blood increases with the support of the released isoforms.

Abrahao *et al* .^18^ compared experimental rat models in the small intestine with IR damage in terms of ischemia and rats that they reperfused with (IPC) in terms of LDH values and could not show any statistical difference between them. They attributed this to the fact that LDH is a nonspecific tissue damage indicator. Mallick *et al* .^16^ , in their studies in which they caused experimental IR damage in the rat small intestine, used an oxygenase precursor prolidine dithiocarbamate as a preconditioning agent and found a significant decrease in serum LDH levels compared to the IR group.

In our study, there was a statistically significant decrease in LDH levels compared to the control group in the PPC group in which both IPC and BK were used (p <0.001).

In the postischemic state, PMNL also causes the formation of free oxygen radicals that play a role in IR damage with the MPO enzyme they contain. Grisham *et al* .^28^ have shown that reperfusion in the cat intestine causes an 18-fold increase in mucosal MPO levels with PMN accumulation. Ferrer *et al.* ^29^ found significant decreases in tissue MPO levels by removing the effect of free radicals in the rat intestinal IR model, in which they used Allopurinol as antioxidants^29^ . Özden *et al* .^30^ using anti-thrombin III, examined its effects on lipid peroxidation in intestinal IR damage and detected significant MPO reductions by inhibiting lipid peroxidation to some extent.

In our study, there was a 2.5-fold increase in MPO activity in the control group compared to the sham group. Statistically, this difference is significant (p <0.001). This result reflects the damage to the intestinal mucosa by reperfusion. The highest MPO values are still in the control group. Statistically significant low tissue MPO levels were detected in both the ipc and ppc groups compared to the control group (p <0.001).

Many scoring systems have been developed for histopathological evaluation of intestinal IR damage. Histopathological classification as defined by Intestinal IR injury and the effect of the preconditioning agents used against it is a frequently used and simple morphological scoring system that can be evaluated histopathologically.

Mallick *et al* .^16^ used prolidin dithiocarbamate as a preconditioning agent in their studies in which they caused experimental IR damage in the rat small intestine and examined the intestinal morphology changes according to the scoring system described by Chiu *et al* .^31^ They found a significant decrease in the histopathological score compared to the IR group in the study group^16^ . Abrahao *et al* .^18^ in their experimental studies evaluating the protective effects of IP against intestinal IR damage in terms of biochemical and morphology, did not show a statistical difference between the IR group and the IPC group.

In our study, in the group in which we created experimental IR damage, shedding in villi, increased cellularity in lamina propria, mucosal ulceration and necrosis were observed. These histopathological findings reflect advanced reperfusion injury according to the Chiu scoring^31^ . In the study groups, capillary congestion and widespread epithelial separation were observed in the upper parts of the villi. When the groups were evaluated in terms of histopathological damage, it was seen that the most damage was in the control group. Significant improvement was observed in histopathological changes with the application of IPC and / or BK. However, there was no statistically significant difference between these two study groups.

As a result of this study, it was understood by using biochemical and morphological parameters that both IPC and PPC using BK were effective methods to reduce IR damage frequently encountered during small bowel surgery but they did not have superiority to each other.

BK is produced endogenously by tissues in ischemic preconditioning mechanisms and contributes to protective mechanisms^32^ . From this point of view, we preferred to use BK as a preconditioning agent by giving it externally as a pharmacological agent. BK can protect the intestinal mucosa from reperfusion damage by inhibiting neutrophil adhesion in addition to activating endogenous protective mechanisms.

## Conclusions

The potent vasodilator effect of BK may counterbalance vasoconstriction, a prominent mechanism in reperfusion injury. As a matter of fact, we achieved similar results with BK compared to IPC, which has previously proven efficacy in the small intestine.

Given the interrelated and highly complex mechanisms of reperfusion injury, studies are needed to elucidate the different mechanisms of action in the small intestine with BK, and in particular studies using other parameters.

## Footnotes

1 Research performed at Hakan Çetinsaya Experimental and Clinical Research Center (DEKAM), Faculty of Medicine, Erciyes University, Melikgazi, Kayseri, Turkey.
